# Supplementary material for: Integrated analysis of the aqueous humor microbiome and lens capsule transcriptome in high myopia cataract: a pilot study
Source: Front Med (Lausanne). 2026 Jun 16;13:1845205. doi: 10.3389/fmed.2026.1845205 (PMC13314463; doi:10.3389/fmed.2026.1845205)
Supplement: Supplementary file 3 [file Data_Sheet_1.zip › supplemental_material/EN_method.pdf]

注：Method 描述模板仅供参考，请根据实际情况修改使用。(v202402)

Library construction and sequencing

The 2bRAD-M library preparation basically followed the original protocol developed by Wang et al. [1] with minor modifications. DNA (1 pg-200 ng) was digested with 4 U of the enzyme BcgI (NEB) for 3 h at 37 ℃. Subsequently, the adaptors were ligated to the DNA fragments. The ligation reaction was performed by combining 10 μl of digested DNA with 10 μl of a ligation master mix containing 0.2 μM each of two adaptors and 800 U T4 DNA ligase (NEB). Ligation was carried out at 4 ℃ for 12 h. Then, ligation products were amplified, and PCR products were subjected to 8% polyacrylamide gel. Bands of approximately 100 bp were excised from the polyacrylamide gel, and the DNA was diffused from the gel in nuclease-free water for 6-12 h at 4 ℃. Sample-specific barcodes were introduced by PCR with platform-specific barcode-bearing primers. Each 20 μl PCR contained 6 ul of gel-extracted PCR product, 0.2 μM of each primer, 0.3 mM dNTP, 1×Phusion HF buffer and 0.4 U Phusion high-fidelity DNA polymerase (NEB). PCR products were purified using QIAquick PCR purification kit (Qiagen) and then subjected to sequencing using the Illumina Nova PE150 platform. 2bRAD-M was carried out at the Qingdao OE Biotech Co.,Ltd. (Qingdao, China).

Data analysis

1. **Identification of species-specific 2bRAD-M markers from the most comprehensive genome database**

Firstly, totally 404,199 microbial genomes (including bacteria, fungi and archaea) were downloaded from GTDB and Ensembl database. Then, built-in Perl scripts were used to sample restriction fragments from microbial genomes by each of 16 type 2B restriction enzymes, which formed a huge 2bRAD microbial genome database. The set of 2bRAD tags sampled from each genome was assigned under the GCF number, as well as GCF's taxonomic information corresponding to the whole genome[2,3].

Finally, all 2bRAD tags from each GCF that occur once within the genome were compared

with those to all the others. Those 2bRAD tags are specific to a species-level taxon (having no overlap with other species) were developed as species-specific 2bRAD markers, collectively forming a 2bRAD marker database.

2. Calculation of relative abundance

Firstly, to identify microbial species within each sample, all sequenced 2bRAD tags after quality control were mapped (using a built-in Perl script) against the 2bRAD marker database which contains all 2bRAD tags theoretically unique to each of 86,022 microbial species in database. To control the false-positive in the species identification, G score was derived for each species identified within a sample as below, which is a harmonious mean of read coverage of 2bRAD markers belongs to a species and number of all possible 2bRAD markers of this species. The threshold of G score for a false positive discovery of microbial species was set 5[3].

$$\text{G}\ score_{\ species\ i} = \sqrt{S_i \times t_i}$$

S: the number of reads assigned to all 2bRAD markers belonging to species i within a sample.

t: number of all 2bRAD markers of species i that have been sequenced within a sample.

Then, calculated the average read coverage of all 2bRAD markers for each species, which represent the number of individuals belonging to a species present in a sample at a given sequencing depth. The relative abundance of a given species is then calculated as the ratio of the number of microbial individuals belonging to a species against the total number of individuals from known species that can be detected within a sample.

$$Relative\ abundance_{\ species\ i} = \frac{S_i/T_i}{\sum_{i=1}^{n} S_i/T_i}$$

S: the number of reads assigned to all 2bRAD markers of species i within a sample.

T: the number of all theoretical 2bRAD markers of species i.

References（请注意引用参考文献）

[1] Wang, Shi, et al. "2b-RAD: a simple and flexible method for genome-wide genotyping." *Nature methods* 9.8 (2012): 808-810.

[2] Sun, Zheng, et al. "Removal of false positives in metagenomics-based taxonomy profiling via targeting Type IIB restriction sites." *Nature Communications* 14.1 (2023): 5321.

[3] Sun, Zheng, et al. "Species-resolved sequencing of low-biomass or degraded microbiomes using 2bRAD-M." *Genome biology* 23.1 (2022): 36.
